# An SDP-based approach for computing the stability number of a graph

**DOI:** 10.1007/s00186-022-00773-1

**Published:** 2022-03-12

**Authors:** Elisabeth Gaar, Melanie Siebenhofer, Angelika Wiegele

**Affiliations:** 1 Institute of Production and Logistics Management, Johannes Kepler University Linz, Altenberger Straß 69, 4040 Linz, Austria; 2 Institut für Mathematik, Alpen-Adria-Universität Klagenfurt, Universitätsstraße 65-67, 9020 Klagenfurt, Austria

**Keywords:** Stable set, Semidefinite programming, Lovász theta function, Branch and bound, Combinatorial optimization

## Abstract

Finding the stability number of a graph, i.e., the maximum number of vertices of which no two are adjacent, is a well known NP-hard combinatorial optimization problem. Since this problem has several applications in real life, there is need to find efficient algorithms to solve this problem. Recently, Gaar and Rendl enhanced semidefinite programming approaches to tighten the upper bound given by the Lovász theta function. This is done by carefully selecting some so-called exact subgraph constraints (ESC) and adding them to the semidefinite program of computing the Lovász theta function. First, we provide two new relaxations that allow to compute the bounds faster without substantial loss of the quality of the bounds. One of these two relaxations is based on including violated facets of the polytope representing the ESCs, the other one adds separating hyperplanes for that polytope. Furthermore, we implement a branch and bound (B&B) algorithm using these tightened relaxations in our bounding routine. We compare the efficiency of our B&B algorithm using the different upper bounds. It turns out that already the bounds of Gaar and Rendl drastically reduce the number of nodes to be explored in the B&B tree as compared to the Lovász theta bound. However, this comes with a high computational cost. Our new relaxations improve the run time of the overall B&B algorithm, while keeping the number of nodes in the B&B tree small.

## Introduction

The stable set problem is a fundamental combinatorial optimization problem. It is capable of modeling other combinatorial problems as well as real-world applications and is therefore widely applied in areas like operations research or computer science. We refer to the survey (Wu and Hao 2015) for more information and a review of exact and heuristic algorithms. Most of the exact algorithms are based on branch and bound (B&B) and differ mainly by different upper and lower bound computations. A recent paper using a MIP solver is e.g. (Letchford et al. 2020). The models used in that paper yield computation times from less than a second up to half an hour on a selection of DIMACS instances.

Outstanding results are obtained by an algorithm of Depolli et al. (2013). They introduced an algorithm using parallel computing for finding maximum cliques in the context of protein design. The algorithm consists of carefully implemented algorithmic building blocks such as an approximate coloring algorithm, an initial vertex ordering algorithm and the use of bit-strings for encoding the adjacency matrix.

In the 2015 survey (Wu and Hao 2015), no exact algorithms using semidefinite programming (SDP) are mentioned. One reason for the rare literature on SDP based B&B algorithms is the high computational cost for computing these bounds. In this work we introduce an SDP based B&B algorithm. We formulate new SDP relaxations and develop solution algorithms to compute these bounds with moderate computational expense, making them applicable within a B&B scheme.

Before introducing the stable set problem, sometimes also referred to as vertex packing problem, we give the definition of a stable set. Let $G = (V,E)$ be a simple undirected graph with $|V| = n$ vertices and $|E| = m$ edges. A set $S \subseteq V$ is called stable if no vertices in *S* are adjacent. *S* is called a maximal stable set if it is not possible to add a vertex to *S* without losing the stability property. The stability number $\alpha \left( G\right)$ denotes the maximum size of a stable set in *G*, where size means the cardinality of the set. A stable set *S* is called a maximum stable set if it has size $\alpha (G)$.

For convenience, from now on we always label the vertices of a graph with *n* vertices from 1 to *n*. Computing $\alpha (G)$ can be done by solving the following optimization problem.

1 $$\begin{aligned} \alpha (G) =&\max \qquad \sum _{i=1}^{n} x_i \\ \nonumber&{{\,\mathrm{s.t.}\,}}x_i + x_j \le 1 ~\forall \{i,j\} \in E(G) \\ \nonumber&x \in \{0,1\}^n \end{aligned}$$

For a graph $G = (V,E)$, the set of all stable set vectors $\mathcal {S}(G)$ and the stable set polytope ${{\,\mathrm{STAB}\,}}(G)$ are defined as

$$\begin{aligned} \mathcal {S}(G)&= \left\{ s \in \{0,1\}^{n} : s_{i}s_{j} = 0 \quad \forall \{i,j\} \in E\right\} \text { and}\\ {{\,\mathrm{STAB}\,}}(G)&= {{\,\mathrm{conv}\,}}\left\{ s : s \in \mathcal {S}(G)\right\} . \end{aligned}$$

Determining $\alpha (G)$ is NP-complete and the decision problem is among Karp’s 21 NP-complete problems (Karp 1972). Furthermore, Håstad (1999) proved that $\alpha (G)$ is not approximable within $n^{1{-}\varepsilon }$ for any $\varepsilon > 0$ unless P=NP. A well known upper bound on $\alpha (G)$ is the Lovász theta function $\vartheta (G)$. Grötschel et al. (1988) proved that

2 $$\begin{aligned} \vartheta (G) =&\max \qquad \mathbbm {1}_{n}^{T}x \\ \nonumber&{{\,\mathrm{s.t.}\,}}{{\,\mathrm{diag}\,}}(X) = x\\ \nonumber&\qquad \quad \qquad X_{i,j} = 0\,\,\,\quad \forall \{i,j\} \in E \\ \nonumber&\qquad \left( \begin{array}{cc} 1 &{} x^{T} \\ x &{} X \end{array}\right) \succcurlyeq 0\\ \nonumber&\qquad X \in {\mathcal S}_{n}\quad ,~x \in \mathbb {R}^{n} \end{aligned}$$

and hence provided a semidefinite program (SDP) to compute $\vartheta (G)$. We define the feasible region of (2) as

$$\begin{aligned} {{\,\mathrm{TH}\,}}^{2}(G)&= \left\{ (x,X) \in \mathbb {R}^{n}\times {\mathcal S}_{n} :{{\,\mathrm{diag}\,}}(X) = x, \right. \\&\left. \qquad \qquad X_{i,j}=0 \quad \forall \{i,j\} \in E, \quad X-xx^{T} \succcurlyeq 0 \right\} . \end{aligned}$$

Clearly for each element (*x*, *X*) of ${{\,\mathrm{TH}\,}}^{2}(G)$ the projection of *X* onto its main diagonal is *x*. The set of all projections

$$\begin{aligned} {{\,\mathrm{TH}\,}}(G) = \left\{ x \in \mathbb {R}^{n}:\exists X \in {\mathcal S}_{n} : (x,X) \in {{\,\mathrm{TH}\,}}^{2}(G) \right\} \end{aligned}$$

is called theta body. More information on ${{\,\mathrm{TH}\,}}(G)$ can be found for example in Conforti et al. (2014). It is easy to see that ${{\,\mathrm{STAB}\,}}(G) \subseteq {{\,\mathrm{TH}\,}}(G)$ holds for every graph *G*, see (Grötschel et al. 1988). Thus $\vartheta (G)$ is a relaxation of $\alpha (G)$.

This paper is structured as follows. In Sect. 2 we introduce two new relaxations using the concept of exact subgraph constraints. A branch and bound algorithm that uses these relaxations is described in Sect. 3, followed by the discussion of numerical results in Sect. 4. Sect. 5 concludes this paper.

## New relaxations of the exact subgraph constraints

In this section we present two new approaches to find upper bounds on the stability number $\alpha (G)$ of a graph *G* starting from the Lovász theta function $\vartheta (G)$ with so-called exact subgraph constraints, one based on violated facets and one based on separating hyperplanes. After introducing these approaches, we compare them both theoretically and practically.

### Basic setup for exact subgraph constraints

Our approach is based on the idea of exact subgraph constraints that goes back to Adams, Anjos, Rendl and Wiegele (Adams et al. 2015) for combinatorial optimization problems that have an SDP relaxation and was recently computationally investigated by Gaar and Rendl (2019, 2020) for the stable set, the Max-Cut and the coloring problem as a basis. Starting from this, we present two relaxations of including exact subgraph constraints into the SDP for calculating $\vartheta (G)$ that are computationally more efficient.

We first recapitulate the basic concepts of exact subgraph constraints with the notation from Gaar (2020). An upper bound on $\alpha (G)$ is given by the Lovász theta function $\vartheta (G)$. Due to the SDP formulation (2) it can be computed in polynomial time. Adams, Anjos, Rendl and Wiegele (Adams et al. 2015) proposed to improve $\vartheta (G)$ as an upper bound by adding so-called exact subgraph constraints. These exact subgraph constraints can be used to strengthen SDP relaxations of combinatorial optimization problems with a certain property by including subgraph information. For the stable set problem we need the following definitions in order to introduce the exact subgraph constraints. For a graph *G* the squared stable set polytope ${{\,\mathrm{STAB}\,}}^{2}(G)$ is defined as

$$\begin{aligned} {{\,\mathrm{STAB}\,}}^{2}(G) = {{\,\mathrm{conv}\,}}\left\{ ss^{T}: s \in \mathcal {S}(G)\right\} \end{aligned}$$

and matrices of the form $ss^{T}$ for $s \in \mathcal {S}(G)$ are called stable set matrices. Let $G_I$ denote the subgraph induced by the vertex set $I \subseteq V(G)$ with $|I| = k_{I}$. With $X_I$ we denote the submatrix of *X* that results when we delete each row and column corresponding to a vertex that is not in *I*. In other words, $X_I$ is the submatrix of *X* where we only choose the rows and columns corresponding to the vertices in *I*. Then the constraint that asks the submatrix $X_I$ of (2) for an induced subgraph $G_I$ to be in the squared stable set polytope $\text {STAB}^2(G_I)$ is called exact subgraph constraint (ESC).

The *k*-th level of the exact subgraph hierarchy introduced in Adams et al. (2015) is the Lovász theta function (2) with additional ESC for each subgraph of order *k*. In Gaar and Rendl (2019, 2020) this hierarchy is exploited computationally by including the ESC only for a set *J* of subgraphs and then considering

3 $$\begin{aligned} z_{J}(G)= \max \left\{ \mathbbm {1}_{n}^{T}x: (x,X) \in {{\,\mathrm{TH}\,}}^2(G),~ X_{I}\in {{\,\mathrm{STAB}\,}}^{2}(G_{I}) \quad \forall I \in J \right\} . \end{aligned}$$

Clearly, $\alpha (G) \leqslant z_{J}(G)$ holds for every set *J* of subsets of *V*(*G*), so $z_{J}(G)$ is an upper bound on $\alpha (G)$. One of the key remaining questions is how to solve (3). We will compare different implementations and relaxations of this problem in the rest of the paper and start by considering existing methods.

The most straightforward way to solve (3) is to include the ESCs in a convex hull formulation as presented in Gaar and Rendl (2019, 2020); Gaar (2020). We now recall the basic features and follow the presentation from Gaar (2020). As the ESC for a subgraph $G_{I}$ makes sure that $X_{I}\in {{\,\mathrm{STAB}\,}}^{2}(G_{I})$ holds and the polytope ${{\,\mathrm{STAB}\,}}^{2}(G_{I})$ is defined as the convex hull of the stable set matrices, the most intuitive way to formulate the ESC is as a convex combination. Towards that end, for a subgraph $G_{I}$ of *G* induced by the subset $I \subseteq V$, let $|\mathcal {S}(G_{I})| = t_{I}$ and let $\mathcal {S}(G_{I}) = \left\{ s^{I}_{1}, \dots , s^{I}_{t_{I}}\right\}$. Then the *i*-th stable set matrix $S^{I}_{i}$ of the subgraph $G_{I}$ is defined as $S^{I}_{i}= s^{I}_{i}(s^{I}_{i})^{T}$. As a result, the ESC $X_{I}\in {{\,\mathrm{STAB}\,}}^{2}(G_{I})$ can be rewritten as

$$\begin{aligned} X_{I}\in {{\,\mathrm{conv}\,}}\left\{ S^{I}_{i}: 1 \leqslant i \leqslant t_{I}\right\} \end{aligned}$$

and it is natural to implement the ESC for the subgraph $G_{I}$ as

$$\begin{aligned} X_{I}= \sum _{i=1}^{t_{I}} [\lambda _{I}]_{i}S^{I}_{i}, \quad \lambda _{I}\in {\Delta }_{t_{I}}, \end{aligned}$$

where ${\Delta }_{t_{I}}$ is the $t_{I}$-dimensional simplex.

This means that when including the ESC for the subgraph $G_{I}$ into (2) we have $t_{I}$ additional non-negative variables, one additional linear equality constraint for $\lambda _{I}$ and the matrix equality constraint which couples $X_{I}$ and $\lambda _{I}$. We denote the number of equality constraints that are induced by the matrix equality constraint by $b_{I}$ and note that $$b_{I}\leqslant \left( {\begin{array}{c}k_{I}+1\\ 2\end{array}}\right) $$ holds. With this formulation (3) can equivalently be written as

4 $$\begin{aligned} z_J^{C}(G)= \max \left\{ \mathbbm {1}_{n}^{T}x: (x,X) \in {{\,\mathrm{TH}\,}}^2(G),~ X_{I}= \sum _{i=1}^{t_{I}} [\lambda _{I}]_{i}S^{I}_{i}, \quad \lambda _{I}\in {\Delta }_{t_{I}} \quad \forall I \in J \right\} ,\nonumber \\ \end{aligned}$$

so $$ z_{J}(G)= z_J^{C}(G)$$ holds. In practice, this SDP can be solved by interior point methods as long as the number of ESC constraints is of moderate size.

Due to the fact that (4) becomes a huge SDP as soon as the number of ESCs |*J*| becomes large, Gaar and Rendl (2019, 2020) proposed to use the bundle method to solve this SDP. The bundle method is an iterative procedure to find a global minimum of a non-smooth convex function and has been adapted for SDPs by Helmberg and Rendl (2000). As we use the bundle method only as a tool and do not enhance it any further, we refrain from presenting details here.

### Relaxation based on inequalities that represent violated facets

We will see later on that the computational costs of a B&B algorithm are enormous in the original version with the convex hull formulation (4) and they are still substantial with the bundle approach from Gaar and Rendl (2019, 2020). Therefore, we suggest two alternatives.

First, we present a relaxation of calculating the Lovász theta function with ESCs (3) that has already been mentioned in Gaar (2020), but has never been computationally exploited so far. The key ingredient for this relaxation is the following observation. The polytope ${{\,\mathrm{STAB}\,}}^{2}(G_{I})$ is given by its extreme points, which are the stable set matrices of $G_{I}$. Due to Weyl’s theorem (see for example (Nemhauser and Wolsey 1988)) it can also be represented by its facets. This means that there are (finitely many) inequalities, such that the constraint $X_{I}\in {{\,\mathrm{STAB}\,}}^{2}(G_{I})$ can be represented by these inequalities.

However, the facets and hence the inequalities depend on the stable set matrices and therefore on the subgraph $G_{I}$. Thus different subgraphs need different calculations that will lead to different inequalities. Gaar (2020)[Lemma 3] showed that adding the ESC $X_{I}\in {{\,\mathrm{STAB}\,}}^{2}(G_{I})$ to the SDP calculating the Lovász theta function (2) is equivalent to adding the constraint $X_{I}\in {{\,\mathrm{STAB}\,}}^{2}(G^{0}_{k_{I}})$ where $G^{0}_{k_{I}} = (V^{0}_{k_{I}},E^{0})$ with $V^{0}_{k_{I}} = \{1, \dots , k_{I}\}$ and $E^{0}= \emptyset$.

This implies that it is enough to calculate the facets of ${{\,\mathrm{STAB}\,}}^{2}(G^{0}_{k_{I}})$ and include these facets for each subgraph $G_{I}$ on $k_{I}$ vertices, instead of calculating the facets of ${{\,\mathrm{STAB}\,}}^{2}(G_{I})$ for each subgraph $G_{I}$ separately. Let $r_{k_{I}}$ be the number of facets of ${{\,\mathrm{STAB}\,}}^{2}(G^{0}_{k_{I}})$ and let $F^{k_{I}}_{i} \in \mathbb {R}^{k_{I}\times k_{I}}$, $f^{k_{I}}_{i} \in \mathbb {R}$ for $1 \leqslant i \leqslant r_{k_{I}}$ such that

$$\begin{aligned} {{\,\mathrm{STAB}\,}}^{2}(G^{0}_{k_{I}}) = \left\{ X \in \mathbb {R}^{k_{I}\times k_{I}}: \left\langle F^{k_{I}}_{i}, X \right\rangle \leqslant f^{k_{I}}_{i} \quad \forall 1 \leqslant i \leqslant r_{k_{I}} \right\} , \end{aligned}$$

so $(F^{k_{I}}_{i},f^{k_{I}}_{i})$ is an inequality representing the *i*-th facet of ${{\,\mathrm{STAB}\,}}^{2}(G^{0}_{k_{I}})$. We obtained $r_{k_{I}}$, $F^{k_{I}}_{i}$ and $f^{k_{I}}_{i}$ for $k_{I}\leqslant 6$ in the way suggested in Gaar (2020). For $k_{I}\geqslant 7$ this computation is beyond reach, as $r_{7}$ is conjectured to be 217093472 (Christof (2020)).

If we would include all facets of ${{\,\mathrm{STAB}\,}}^{2}(G^{0}_{k_{I}})$ for each subgraph $G_{I}$ to replace the ESCs in (3), then we would include a huge number of inequalities ($r_{5} = 368$ and $r_{6}= 116764$) and reach the limits of computing power rather soon. In order to reduce the number of inequalities, for each subgraph we include only those inequalities that represent facets that are violated by the current solution $X^{*}$. To be more precise, let $X^{*}$ be the optimal solution of (3) for $J = \emptyset$, i.e., the optimal solution of calculating the Lovász theta function. Then we define the indices of significantly violated facets of $G_{I}$, i.e., facets where the corresponding inequalities are violated at least by $\varepsilon _{F}$, as

$$\begin{aligned} \mathcal {V}_{I}'= \left\{ 1 \leqslant i \leqslant r_{k_{I}}: \left\langle F^{k_{I}}_{i}, X_{I}^{*}\right\rangle > f^{k_{I}}_{i} + \varepsilon _{F}\right\} , \end{aligned}$$

where $\varepsilon _{F}$ is a small constant to take care of numerical inaccuracies of calculating $X^{*}$.

Now we can further reduce the number of included inequalities in the following way. Although all $(F^{k_{I}}_{i},f^{k_{I}}_{i})$ are different for different values of *i*, it could happen that for a subgraph $G_{I}$ there exist $1 \leqslant i \ne i' \leqslant r_{k_{I}}$ such that $(F^{k_{I}}_{i},f^{k_{I}}_{i})$ and $(F^{k_{I}}_{i'},f^{k_{I}}_{i'})$ induce the same inequality. This is possible because they might differ only in positions $(j,j')$ with $j,j' \in I$ and $\{j,j'\} \in E$. Therefore, these different entries are multiplied with zero due to $[X_{I}^{*}]_{j,j'} = 0$. Hence, let $\mathcal {V}_{I}\subseteq \mathcal {V}_{I}'$ be a set such that only one index among all indices in $\mathcal {V}_{I}'$ which induce the same inequality is in $\mathcal {V}_{I}$. Then we obtain the following relaxation of (3), in which we include only inequalities that induce significantly violated facets of $G_{I}$

5 $$\begin{aligned} z_J^{F}(G)= \max \left\{ \mathbbm {1}_{n}^{T}x: (x,X) \in {{\,\mathrm{TH}\,}}^2(G),~ \left\langle F^{k_{I}}_{i}, X_{I}\right\rangle \leqslant f^{k_{I}}_{i} \quad \forall i \in \mathcal {V}_{I}\quad \forall I \in J \right\} .\nonumber \\ \end{aligned}$$

Unfortunately for $k_{I}\geqslant 7$ it is not possible to store and check the facets of ${{\,\mathrm{STAB}\,}}^{2}(G^{0}_{k_{I}})$ for violation in reasonable memory and time due to the huge number of facets. Hence, we can perform this relaxation only for subgraphs $G_{I}$ of order $k_{I}\leqslant 6$.

### Relaxation based on separating hsyperplanes

Next we consider another approach to implement a relaxation of (3) which can also be used for subgraphs $G_{I}$ of order $k_{I}\geqslant 7$ and which is based on including separating hyperplanes.

It uses the following fact. Let $\tilde{X}$ be any matrix in $\in {\mathcal S}_{n}$ and let $P_{I}$ be the projection of $\tilde{X}_{I}$ onto ${{\,\mathrm{STAB}\,}}^{2}(G_{I})$. Then we can calculate the projection distance of $\tilde{X}$ to ${{\,\mathrm{STAB}\,}}^{2}(G_{I})$ as

6 $$\begin{aligned} \left\Vert P_{I}- \tilde{X}_{I} \right\Vert _{F}^2\nonumber&= \min _{\lambda _{I}\in {\Delta }_{t_{I}}} \left\Vert \left( \sum _{i = 1}^{t_{I}} [\lambda _{I}]_{i}S^{I}_{i}\right) - \tilde{X}_{I} \right\Vert _{F}^2\nonumber = \min _{\lambda _{I}\in {\Delta }_{t_{I}}} \left\Vert \sum _{i = 1}^{t_{I}} [\lambda _{I}]_{i}(S^{I}_{i}- \tilde{X}_{I}) \right\Vert ^{2}_{F}\nonumber \\&= \min _{\lambda _{I}\in {\Delta }_{t_{I}}} \sum _{j = 1}^{k_{I}} \sum _{j' = 1}^{k_{I}} \left( \sum _{i = 1}^{t_{I}} [\lambda _{I}]_{i} \left[ S^{I}_{i}- \tilde{X}_{I}\right] _{j,j'}\right) ^{2}\nonumber \\&= \min _{\lambda _{I}\in {\Delta }_{t_{I}}} \sum _{j = 1}^{k_{I}} \sum _{j' = 1}^{k_{I}} \left( \sum _{i = 1}^{t_{I}} \sum _{i'=1}^{t_{I}} [\lambda _{I}]_{i}[\lambda _{I}]_{i'} \left[ S^{I}_{i}- \tilde{X}_{I}\right] _{j,j'} \left[ S^{I}_{i'}- \tilde{X}_{I}\right] _{j,j'} \right) \nonumber \\&= \min _{\lambda _{I}\in {\Delta }_{t_{I}}} \sum _{i = 1}^{t_{I}} \sum _{i'=1}^{t_{I}} [\lambda _{I}]_{i}[\lambda _{I}]_{i'} \left( \sum _{j = 1}^{k_{I}} \sum _{j' = 1}^{k_{I}} \left[ S^{I}_{i}- \tilde{X}_{I}\right] _{j,j'} \left[ S^{I}_{i'}- \tilde{X}_{I}\right] _{j,j'} \right) \nonumber \\&= \min _{\lambda _{I}\in {\Delta }_{t_{I}}} \lambda _{I}^{T} Q_{I}\lambda _{I}, \end{aligned}$$

where $Q_{I}\in \mathbb {R}^{t_{I}\times t_{I}}$ and $[Q_{I}]_{i,i'} = \left\langle S^{I}_{i}- \tilde{X}_{I}, S^{I}_{i'}- \tilde{X}_{I}\right\rangle$. $Q_{I}$ is symmetric and positive semidefinite because it is a Gram matrix, so (6) is a convex-quadratic program with $t_{I}$ variables, a convex-quadratic objective function and one linear equality constraint. With the optimal solution $\lambda _{I}$ of (6) the projection of $\tilde{X}_{I}$ onto ${{\,\mathrm{STAB}\,}}^{2}(G_{I})$ can be obtained by $P_{I}= \sum _{i = 1}^{t_{I}} [\lambda _{I}]_{i}S^{I}_{i}$. By defining

$$\begin{aligned} H_{I}= \frac{1}{\left\Vert \tilde{X}_{I}- P_{I} \right\Vert _{F}} \left( \tilde{X}_{I}- P_{I}\right) \quad \text { and } \quad h_{I}= \frac{1}{\left\Vert \tilde{X}_{I}- P_{I} \right\Vert _{F}} \left\langle \tilde{X}_{I}- P_{I}, P_{I}\right\rangle \end{aligned}$$

due to the separating hyperplane theorem (see for example Boyd and Vandenberghe 2004)

7 $$\begin{aligned} \left\langle H_{I}, X_{I}\right\rangle \leqslant h_{I}\end{aligned}$$

is a hyperplane that separates $\tilde{X}_{I}$ from ${{\,\mathrm{STAB}\,}}^{2}(G_{I})$ such that $X_{I}= P_{I}$ fulfills the inequality with equality. Obviously (7) is a relaxation of the ESC $X_{I}\in {{\,\mathrm{STAB}\,}}^{2}(G_{I})$, so

8 $$\begin{aligned} z_J^{H}(G)= \max \left\{ \mathbbm {1}_{n}^{T}x: (x,X) \in {{\,\mathrm{TH}\,}}^2(G),~ \left\langle H_{I}, X_{I}\right\rangle \leqslant h_{I}\quad \forall I \in J \right\} \end{aligned}$$

is another relaxation of (3) that depends on the chosen $\tilde{X}$.

### Theoretical comparison of the relaxations

We briefly comment on some theoretical properties of $z_J^{C}(G)$, $z_J^{F}(G)$ and $z_J^{H}(G)$. We start by analyzing the upper bounds we obtain. Due to the fact that $z_J^{F}(G)$ and $z_J^{H}(G)$ are relaxations of $z_J^{C}(G)$, we know that

$$\begin{aligned} \alpha (G) \leqslant z_J^{C}(G)\leqslant z_J^{F}(G), z_J^{H}(G)\leqslant \vartheta (G) \end{aligned}$$

holds for every graph *G* and every set *J*.

Another important observation is the following. Whenever we include the ESC of the subgraph $G_{I}$ into the SDP computing $z_J^{C}(G)$, the stable set problem is solved exactly on this subgraph $G_{I}$. However, when computing $z_J^{F}(G)$ and $z_J^{H}(G)$ we do not include the ESC but only a relaxed version of it. Hence, in the optimal solutions of these two relaxations, it could still be the case that the ESC is not fulfilled, i.e., for the subgraph $G_{I}$ we do not have an exact solution. Hence, it is possible that we still find violated inequalities (representing facets or hyperplanes) in these cases. As a consequence, for $z_J^{C}(G)$ it does not make sense to include the ESC for the same subgraph twice, but for $z_J^{F}(G)$ and $z_J^{H}(G)$ it is possible that we want to include a relaxation of the very same ESC twice with different facets or a different separating hyperplane.

Finally let us consider the sizes of the SDPs to solve. In all three versions $z_J^{C}(G)$, $z_J^{F}(G)$ and $z_J^{H}(G)$ we solve the SDP of the Lovász theta function (2) with additional constraints, so in all three SDPs we have a matrix variable of dimension $n+1$ which has to be positive semidefinite (psd) and $n+m+1$ linear equality constraints. Additionally to that we have $\sum _{I \in J} t_{I}$ non-negative variables and $|J|+\sum _{I \in J}b_{I}$ equality constraints for $z_J^{C}(G)$, $\sum _{I \in J} |\mathcal {V}_{I}|$ inequalities for $z_J^{F}(G)$, and $|J|$ inequalities for $z_J^{H}(G)$. Table 1 gives an overview of the different sizes of the SDPs.

**Table 1:** Sizes of the SDPs to compute $z_J^{C}(G)$, $z_J^{F}(G)$ and $z_J^{H}(G)$

	$z_J^{C}(G)$	$z_J^{F}(G)$	$z_J^{H}(G)$
Dimension psd matrix variable	$n+1$	$n+1$	$n+1$
# Non-negative variables	$\sum _{I \in J} t_{I}$	0	0
# Linear equality constraints	$n+m+1 + |J|+\sum _{I \in J}b_{I}$	$n+m+1$	$n+m+1$
# Linear inequality constraints	0	$\sum _{I \in J} |\mathcal {V}_{I}|$	$|J|$

### Computational comparison of the relaxations

Before we perform a large scale comparison of $z_J^{C}(G)$, $z_J^{F}(G)$ and $z_J^{H}(G)$ within a B&B algorithm, we investigate a small example.

#### Example 1

We consider a random graph $G=G_{100,15}$ from the Erdős-Rényi model *G*(*n*, *p*) with $n=100$ and $p=0.15$. A random graph from this model has *n* vertices and every edge is present with probability *p* independently from all other edges. For the chosen graph, $\vartheta (G_{100,15}) = 27.2003$ and $\alpha (G_{100,15}) = 24$ holds, so

$$\begin{aligned} 24 \leqslant z_J^{C}(G)\leqslant z_J^{F}(G), z_J^{H}(G)\leqslant 27.2003 \end{aligned}$$

holds for every set *J*.

All the computations were performed on an Intel(R) Core(TM) i7-7700 CPU @ 3.60GHz with 32 GB RAM with the MATLAB version R2016b and with MOSEK version 8. In the computations, we use $\varepsilon _{F}= 0.00005$ and we include a separating hyperplane for a subgraph whenever the projection distance is greater or equal to 0.00005.

We compute $z_J^{C}(G)$, $z_J^{F}(G)$ and $z_J^{H}(G)$ for different sets *J*, which all consist of subsets of vertices of size $k_{I}= 5$ and only differ in the number $q=|J|$ of included ESCs. To be more precise, we consider five different sets $J = J_{q}$ with $q= |J_{q}| \in \{221,443,664,886,1107\}$. These values of $q$ are chosen in such a way that the number of linear equality constraints which are induced by the matrix equalities from the ESCs in the convex hull formulation, i.e., $\sum _{I \in J_{q}} b_{I}$, is in $\{3000,6000,9000,12000,15000\}$. To choose the subsets in $J_{q}$, we first determine $X^{*}$ as the optimal solution of (3) for $J = \emptyset$, i.e., the optimal solution of calculating the Lovász theta function (2). Then we generated $3q$ subgraphs $G_{I}$ of order $k_{I}$ randomly and included those $q$ subsets *I* into $J_{q}$, where the corresponding $X^{*}_I$ have the largest projection distances to ${{\,\mathrm{STAB}\,}}^{2}(G_{I})$. For computing $z_J^{H}(G)$ we choose $\tilde{X}= X^{*}$.

**Table 2:** The values of $z_J^{C}(G)$, $z_J^{F}(G)$ and $z_J^{H}(G)$ for $G=G_{100,15}$ for different sets $J_{q}$

	$J_{221}$	$J_{443}$	$J_{664}$	$J_{886}$	$J_{1107}$
$z_J^{C}(G)$	26.9905	26.9299	26.8684	26.8496	26.8278
$z_J^{F}(G)$	26.9975	26.9393	26.8807	26.8602	26.8397
$z_J^{H}(G)$	27.0104	26.9741	26.9215	26.8992	26.8898

If we consider Table 2 with the improved upper bounds then we see that if $q$ increases, all upper bounds $z_J^{C}(G)$, $z_J^{F}(G)$ and $z_J^{H}(G)$ improve. Furthermore, one can observe that for a fixed set $J_{q}$ the obtained bounds of $z_J^{C}(G)$ are best, those of $z_J^{F}(G)$ are a little bit worse and those of $z_J^{H}(G)$ are even a little bit more worse, i.e., empirically the bounds obtained by using $z_J^{F}(G)$ are better than those coming from $z_J^{H}(G)$ in our example.

**Table 3:** The running times in seconds for computing $z_J^{C}(G)$, $z_J^{F}(G)$ and $z_J^{H}(G)$ of Table 2

	$J_{221}$	$J_{443}$	$J_{664}$	$J_{886}$	$J_{1107}$
$z_J^{C}(G)$	8.14	29.30	66.95	139.64	279.11
$z_J^{F}(G)$	0.61	1.15	1.94	2.72	3.81
$z_J^{H}(G)$	0.75	1.25	1.93	2.57	3.34

Next we consider the running times for computing $z_J^{C}(G)$, $z_J^{F}(G)$ and $z_J^{H}(G)$ in Table 3. Here we see that the time it takes so solve $z_J^{C}(G)$ is extremely high and increases drastically if the number of included ESCs gets larger. Both our relaxations $z_J^{F}(G)$ and $z_J^{H}(G)$ reduce the running times enormously. The running times for $z_J^{F}(G)$ and $z_J^{H}(G)$ are comparable, but computing $z_J^{H}(G)$ is slightly faster for including a large number of ESCs as we only include one additional inequality in $z_J^{H}(G)$ whereas we may include several inequalities that represent facets in $z_J^{F}(G)$.

**Table 4:** The average projection distances of $X_{I}$ to ${{\,\mathrm{STAB}\,}}^{2}(G_{I})$ over all $I \in J_{q}$ before (i.e., $X = X^{*}$ is the optimal solution of (2)), and after (i.e., $X \in \{X^{C*}, X^{F*}, X^{H*}\}$ is the optimal solution of $z_J^{C}(G)$, $z_J^{F}(G)$ and $z_J^{H}(G)$) including the ESCs

	$J_{221}$	$J_{443}$	$J_{664}$	$J_{886}$	$J_{1107}$
Before including ESCs	0.03095	0.03014	0.03057	0.02982	0.03032
After computing $z_J^{C}(G)$	0.00005	0.00004	0.00004	0.00004	0.00004
After computing $z_J^{F}(G)$	0.00151	0.00087	0.00115	0.00080	0.00051
After computing $z_J^{H}(G)$	0.00290	0.00256	0.00252	0.00196	0.00183

As a next step we investigate the projection distances. Recall that $X^{*}$ is the optimal solution of calculating the Lovász theta function (2). Let $X^{C*}$, $X^{F*}$ and $X^{H*}$ be the optimal solution of calculating $z_J^{C}(G)$, $z_J^{F}(G)$ and $z_J^{H}(G)$, respectively. In Table 4 we see that the average projection distance of $X^{*}$ is significantly larger than 0 before including the ESCs, so there are several violated ESCs. As soon as the ESCs are included the average projection distance for $X^{C*}$ is almost zero, so the ESCs are almost satisfied. In theory they should all be zero, but as MOSEK is not an exact solver, the optimal solution is subject to numerical inaccuracies. If we turn to $z_J^{F}(G)$, then the projection distances of $X^{F*}_{I}$ are not as close to zero as those for $X^{C*}$, because $z_J^{F}(G)$ is only a relaxation of $z_J^{C}(G)$. Also the average projection distance of $X^{H*}_{I}$ after solving $z_J^{H}(G)$ is greater than the one obtained with $z_J^{F}(G)$. This is in tune with the fact that the upper bounds obtained in the latter case are better for this instance. Furthermore, note that the average projection distances for $X^{F*}_{I}$ and $X^{H*}_{I}$ decrease as $q$ increases. This is not surprising, as more ESCs mean that a bigger portion of the graph is forced into the stable set structure.

**Table 5:** The average number of violated facets $|\mathcal {V}_{I}|$ over all subgraphs $G_{I}$ with $I \in J_{q}$ before and after including the ESCs and computing $z_J^{F}(G)$

	$J_{221}$	$J_{443}$	$J_{664}$	$J_{886}$	$J_{1107}$
Before including ESCs	1.53	1.56	1.57	1.56	1.56
After computing $z_J^{F}(G)$	0.23	0.14	0.16	0.15	0.09

Finally we present in Table 5 the average number of violated facets. As one can see the average number of violated facets before including the ESCs is already very low. This means that we do not include too many inequalities that represent facets in the computation of $z_J^{F}(G)$. Furthermore, the average number of facets that are violated by $X^{F*}$ decreases significantly compared to the average number of violated facets before including the relaxations of the ESCs. This is very encouraging because one could imagine a scenario where we iteratively add violated facets of one subgraph and then the optimal solution violates different facets. However, the computations suggest that this does not happen too often. Like before in Table 4 we see that the more ESCs are included, the more stable set structure is captured and therefore the less facets are violated after including the relaxations of the ESCs. $\bigcirc$

Let us briefly summarize the key points of Example 1. Usually the upper bounds obtained by $z_J^{F}(G)$ are only slightly worse than those of $z_J^{C}(G)$, but the running times are only a fraction. Unfortunately, this approach works only for subgraphs of order at most 6. Also $z_J^{H}(G)$ yields good upper bounds in slightly better running time than $z_J^{F}(G)$, but the bounds are a little bit worse. A major benefit of this approach is that it can be used for subgraphs of any order.

In a nutshell, both $z_J^{F}(G)$ and $z_J^{H}(G)$ are relaxations of $z_J^{C}(G)$ that reduce the running times drastically by worsening the bounds only a little bit. As a result these bounds are very promising for including them into a B&B algorithm for stable set.

## Branch and bound for the stable set problem

The aim of this section is to present our implementation of an exact branch and bound (B&B) algorithm for the stable set problem (1).

### Our branch and bound algorithm

We start by detailing the general setup of our B&B algorithm. Towards this end, keep in mind that in a solution of (1) the binary variable $x_i$ is equal to 1 if vertex *i* is in the stable set, and $x_i = 0$ otherwise.

For our B&B algorithm for the stable set problem we choose a vertex $i \in V(G)$ and divide the optimization problem in a node of the B&B tree into the subproblem where vertex *i* is in the stable set (i.e., set the branching variable $x_i = 1$) and a second subproblem where *i* is not in the stable set (i.e., set the branching variable $x_i = 0$).

It turns out that in each node of the B&B tree the optimization problem is of the form

9 $$\begin{aligned} P(G,c) = c + \max \quad&\sum \limits _{i \in V(G)} x_i \\ \nonumber {{\,\mathrm{s.t.}\,}}\quad&x_i + x_j \le 1 \quad \forall \{i,j\} \in E(G)\\ \nonumber&x_i \in \{0,1\} \quad \ \,\forall i \in V(G) \end{aligned}$$

for some graph *G*, so in each node we have to solve a stable set problem and add a constant term *c* to the objective function value. Indeed, by fixing a branching variable $x_i$ to 0 or 1, we shrink the graph and create subproblems that are again stable set problems of the form (9) but with a smaller graph and some offset *c*. To be more precise, for the subproblem with $x_i = 1$ the objective function value of (1) increases by 1 because there is one more vertex in the stable set. Furthermore, all neighbors of *i* can not be in the maximum stable set because *i* is already in the stable set. So we can set $x_j = 0$ for all $j \in N_G(i)$ if $N_G(i) = \{j \in V(G) \mid \{i,j\} \in E(G) \}$ denotes the set of neighbors of the vertex *i* in *G*. Furthermore, we can delete *i* and all neighbors of *i* in the current graph *G* and search for a maximum stable set in the new graph $G'$ of smaller order, where $G'= G[U']$ is the subgraph of *G* induced by $U' = V(G)\setminus \bigl (\{i\} \cup N_G(i) \bigr )$. Hence, the subproblem to solve in the new node has the form $P(G',c+1)$.

In the subproblem for $x_i = 0$ the vertex *i* is not in the stable set. We can remove the vertex *i* from the graph and search for a maximum stable set in the induced subgraph $G'' = G[U'']$ with $U'' = V(G)\setminus \{i\}$. This boils down to solving $P(G'',c)$ in the new node of the B&B tree.

Note that whenever we delete a vertex *i* from the graph in the branching process, we set the according variable $x_i$ to a fixed value. Consequently, in every node, all vertices of the original graph *G* are either still present, or the value of the variable corresponding to them is implicitly fixed. Furthermore, we exclude all non-feasible solutions by deleting all neighbors in case of setting the branching variable to 1. Hence, every time we set a variable of a vertex to 1 the set of all vertices of which the variable is set to 1 remains stable and we only obtain feasible solutions of (1). Therefore, from a feasible solution of (9) in any node we can determine a feasible solution of (1) with the same objective function value.

The order of the graph to consider in a node shrinks whenever we branch. As a consequence the B&B tree is of finite size. Whenever we reach a node with a suproblem on a graph with less or equal to 23 vertices, we solve the problem by a fast enumeration procedure that can be employed whenever the subproblems become sufficiently small. To do so, we iterate easily—and especially fast—over all subsets of *V* in descending order with an implementation of Hinnant (2011) in .

*Bounds* We do not want to solve the subproblems (9) in each node to optimality, but only compute an upper and a lower bound on the optimal objective function value. This boils down to obtaining bounds on the stability number of the graph considered in (9). We present details on lower bounds obtained by heuristics in Sect. 3.2.

As upper bounds we use the relaxations based on ESCs in four different versions, namely the convex hull formulation or the bundle method as detailed in Sect. 2.1, the violated facets version as described in Sect. 2.2 or the separating hyperplanes version as presented in Sect. 2.3. For choosing the subset *J* of ESCs, we follow the approach of Gaar and Rendl (2020) in our computations and perform several cycles, i.e., iterations of the repeat until loop, of solving (a relaxation of) (3) and then adjusting the set *J*, as illustrated in Algorithm 1.

In particular, we start with $J = \emptyset$, as preliminary computations have shown that carrying over ESCs to subproblems does not pay off, and in each cycle we update *J* depending on the current optimal solution $X^{*}$ of the SDP solved. We remove all previously added ESCs where the associated dual variables of the optimal solution have absolute value less than 0.01. For finding violated subgraphs (i.e., subgraphs for which the ESC does not hold in $X^{*}$) we use the methods presented in Gaar and Rendl (2020), so we use a local search heuristic to find submatrices of $X^{*}$ that minimize the inner product with some matrices. We let the local search heuristic run for 9*n* times and add random subgraphs to obtain 9*n* subgraphs without duplicates. From these subgraphs we add the 3*n* most violated ones (subsets *I* with largest projection distance of $X^{*}_I$ to ${{\,\mathrm{STAB}\,}}^{2}(G_{I})$) to *J*.

To reduce computational effort, we stop cycling as soon as we do not expect to be able to prune within the next 5 cycles assuming that the decrease of the upper bound $z^*$ in each future cycle is 0.75 of the average decrease we had in the previous cycles. 
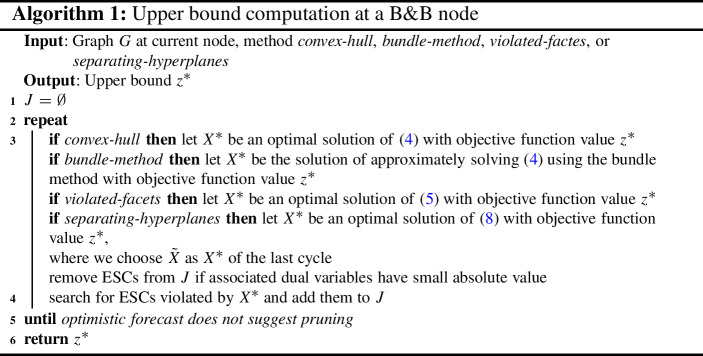


For our computations we use the implementation of the bundle method as it is detailed in Gaar and Rendl (2020), in particular with all specifications given in Section 6.3 therein and we let the bundle method run for at most 15 iterations in each cycle.

*Branching Rule* An important question in the B&B algorithm is how to choose the branching variable. In our implementation we follow the approach to first deal with vertices, for which we know least whether they will be in a maximum stable set or not (*“difficult first”*) in order to find an optimal solution soon.

All our upper bounds are based on the Lovász theta function (2), so we can use the intuition that the closer an entry $x_i$ is to 1 in an optimal solution of (2), the more likely it is that this vertex *i* is in a maximum stable set. Hence, we choose the variable $x_i$ with value closest to 0.5 as branching variable.

More on branching rules for the stable set problem can for example be found in Fondzefe (2016); Xiao (2009).

*Diving Strategy* We implemented a best first search strategy, where we always consider the open subproblem with the largest upper bound next. We expect that we find a large stable set in this branch of the B&B tree because the difference between the global lower bound and the upper bound for this branch is the highest of all.

### Heuristics to find large stable sets

It is crucial to find a good lower bound on $\alpha (G)$ early in the B&B algorithm to prevent the growth of the B&B tree and therefore solve the stable set problem more efficiently. In Singh and Pandey (2015) and Wu and Hao (2015), for example, one can find references to some heuristics to find a large stable set. In our implementation we use several different heuristics.

The first heuristic makes use of the vector *x* from the SDP formulation (2) of $\vartheta (G)$, which is available from the upper bound computation. This vector consists of *n* elements between 0 and 1. The value $x_i$ gives us some intuition about the *i*-th vertex of the graph, namely the closer it is to 1, the more likely it is that the vertex is in a maximum stable set. Hence, we sort the vertices in descending order according to their value in *x* and then add the vertices in this order to a set *S*, such that the vertices of *S* always remain a stable set. In the following we refer to this heuristic as (HT).

Furthermore, we use a heuristic introduced by Khan and Khan (2013) based on vertex covers and vertex supports. A subset *C* of the vertices of a graph is called vertex cover if for each edge at least one of the two incident vertices is in *C*. The vertex support of a vertex is defined as the sum of the degrees of all vertices in the neighborhood of this vertex. If *C* is a vertex cover, then clearly $V(G)\setminus C$ is a stable set, so instead of searching for a maximum stable set we can search for a vertex cover of minimum cardinality. In a nutshell, the heuristic of Khan and Khan (2013) searches for a vertex with maximum vertex support in the neighborhood of the vertices with minimum vertex support. If there is more than one vertex with maximum support, one with maximum degree is chosen. This vertex is added to the vertex cover *C* and all incident edges are removed. The above steps are repeated until there are no edges left in the graph. In the end we obtain a hopefully large stable set with $V(G)\setminus C$. We denote this heuristic by (HVC).

Finally we use a heuristic proposed by Burer et al. (2002). Their heuristic is based on the SDP formulation of the Lovàsz theta function with additional restriction to the matrix variable to be of low rank. With rank one, a local maximizer of the problem yields a maximal stable set, whereas with rank two the stable set corresponding to the local maximizer does not necessarily have to be maximal, but one can escape to a higher local maximizer which corresponds to a maximal stable set. The C source code of this heuristic is online available at Burer and Monteiro (2014). We use this code with the parameters rank set to 2 and the number of so-called escapes set to 1 in a first version and set to 5 in a second version. Both parameter settings are among the choices that were tested in Burer et al. (2002). We will refer to this versions with (H21) and (H25).

In the B&B algorithm we perform the heuristic (H25) in the root node with a time limit of 20 seconds. Then we only perform the heuristics in every third node of the B&B tree. (HT) and (HVC) are very fast, so we let them run in each node we run heuristics. Furthermore, in the first 10 nodes of the B&B tree we perform (H25) with the hope to find a stable set of cardinality $\alpha (G)$ as soon as possible, but we do not allow the heuristic to iterate longer than 7 seconds. For graphs with less than 200 vertices we additionally perform (H21) with the running time limited to 1 second. On graphs with more vertices we perform with probability 0.05 (H25) and a time limit of 7 seconds and in the other cases (H21) with a time limit of 3 seconds. In a computational comparison in the master’s thesis of Siebenhofer (2018), this turned out to be the best combination of heuristics.

## Computational experiments

**Fig. 1:**
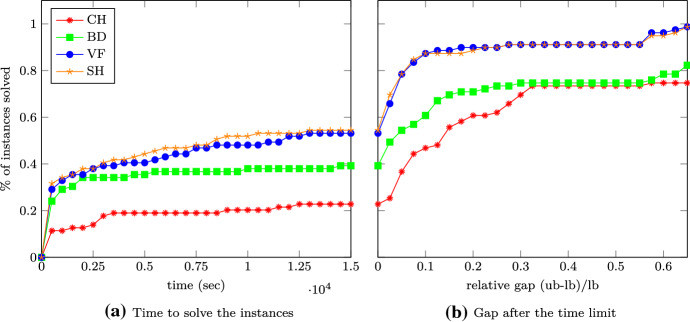
The performance comparison of the bounds (CH), (BD), (VF) and (SH)

In this section we finally compare the B&B algorithms using the different upper bounds presented so far. In Table 6 and Fig. 1 we compare the number of nodes generated in the B&B algorithm as well as the CPU time and the final gap. The abbreviations refer to the following bounds used.

(CH) We consider the upper bound obtained by the ESCs in the convex hull formulation (CH) described in Sect. 2.1,
(BD) solving this formulation with the bundle method (BD) as presented in Sect. 2.1,
(VF) relaxing this formulation by considering only violated facets (VF) as described in Sect. 2.2,
(SH) and using only separating hyperplanes (SH) as presented in Sect. 2.3.
(TH) For better comparability we also consider our B&B algorithm with only the Lovász theta function (2) as an upper bound and denote this version with (TH). Note that this boils down to solving (3) with $J=\emptyset$.

If we are not able to solve an instance within the timelimit, we indicate this in Table 6 by a cell that is colored 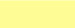. Whenever a cell is colored 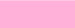 it means that the run did not finish correctly, for example because MOSEK produced an error or ran out of memory. Before discussing the results, we give the details on the instances as well as on the soft- and hardware.

### Benchmark set and experimental setup

We consider several different instances. First, we consider the instances used in Gaar and Rendl (2020), i.e., torus graphs, random near-*r*-regular graphs and random graphs from the Erdős-Rényi model and also several instances from the literature. Additionally we consider all instances from the DIMACS challenge for which the gap between $\vartheta (G)$ and $\alpha (G)$ is larger than one (i.e., all instances which are not solved in the root node of our B&B algorithm) and that have at most 500 vertices. Moreover, we consider spin graphs, which are produced with the command ./rudy -spinglass3pm x x x 50 xxx1 (for $\texttt {x} \in \{5,7,9,11\}$) by the graph generator rudy, which was written by Giovanni Rinaldi.^1^

We implemented our B&B algorithm with different upper bounds in . All computations were performed on an Intel(R) Core(TM) i7-7700 CPU @ 3.60GHz with 32 GB RAM. All programs were compiled with gcc version 5.4.0 with the optimization level -O3 and the CPU time was measured with std::clock. We set the random seed to zero. We use MOSEK [22] 8.1 in the methods (CH), (VF), and (SH) to solve the SDPs (4), (5), and (8), respectively. Furthermore, we use it within the method (BD) for solving the subproblems within the bundle method for computing an approximate solution of (4). Moreover, we use it to solve the QP (6) to compute the projection distance in (SH) and when updating *J*, i.e., while searching for subgraphs with violated ESCs and adding these subgraphs to *J*. The execution time of our B&B algorithm is limited to 4 hours, i.e., after this computation time we allow the B&B to finish solving the SDP of the already started node in the B&B tree and then stop.

**Table 6:**
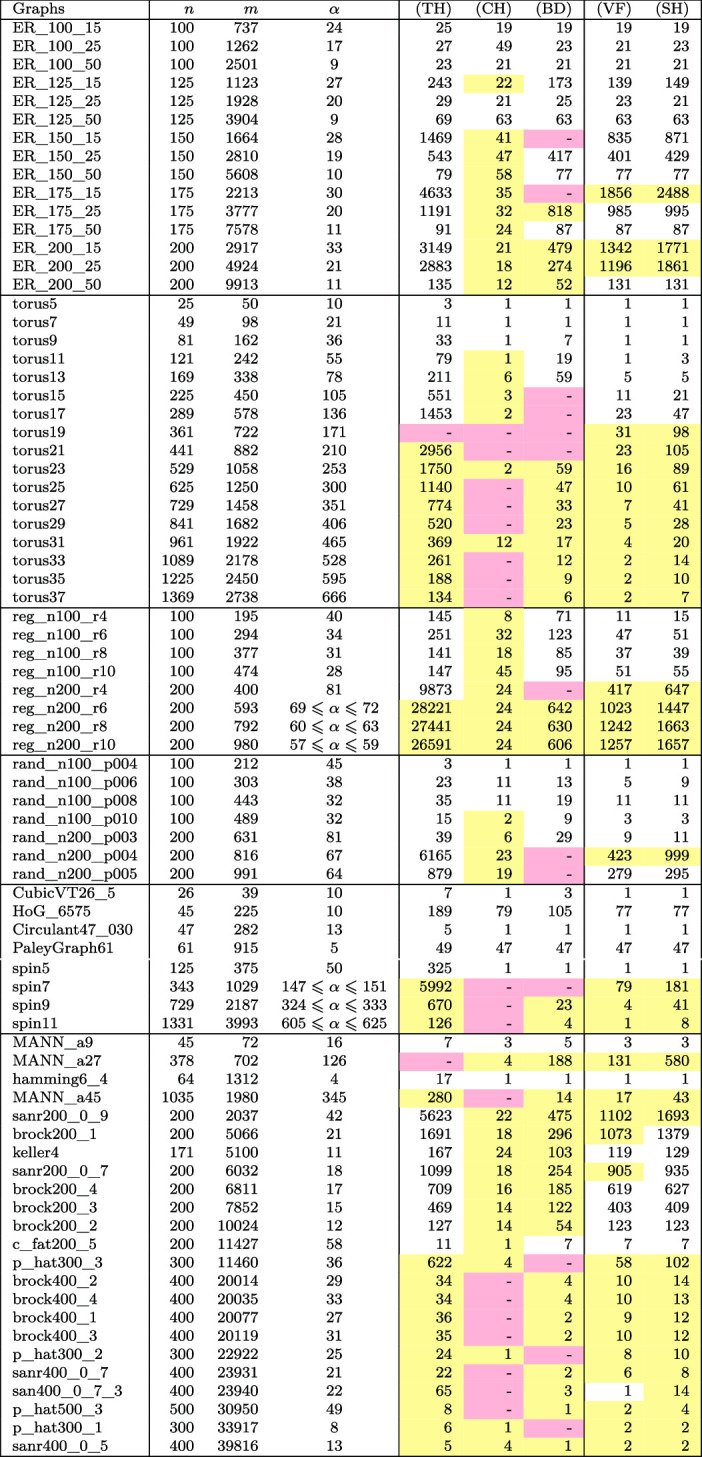
The number of nodes in our B&B algorithm

### First computational experiments

We first want to compare the two different versions of the B&B algorithm that use (CH) and (BD) to compute upper bounds, i.e., we compare those versions that have already been established as upper bounds in the literature, but are now for the first time used within a B&B algorithm.

First, by looking at Table 6 we observe that 20 instances were not solved correctly with (CH), which is due to the fact that the SDPs to solve are huge and therefore MOSEK runs out of memory very often. Indeed, by using (BD) and hence not having to solve that large SDPs only 10 instances are not solved correctly, most of them due to other MOSEK errors. When we compare the number of B&B nodes for the instances where both (CH) and (BD) finished we see that typically the number is the same, or there are slightly more nodes for (BD). This is plausible, as we only have an approximate solution of (3) when using (BD), but an exact solution of (3) with (CH).

We next take a closer look on the lines labeled (CH) and (BD) in the performance profiles in Fig. 1. The B&B code using (BD) as bounding routine can solve much more instances within a given time than when (CH) is used. Once the time limit is reached, the gap is typically much lower for (BD) than it is for (CH).

In a nutshell, even though (BD) solves only a relaxation of (3), using it is much faster than using (CH) while it does not increase the number of B&B nodes a lot. This justifies considering only a relaxation of (3).

### Computational experiments with new relaxations

Up to now we have used the exact subgraph approach of Adams et al. (2015) with the implementation proposed by Gaar and Rendl (2019, 2020) in order to get tight upper bounds on the stability number within a B&B algorithm for solving the stable set problem. So far we have proven the strength of the bounds by showing that the number of nodes in a B&B algorithm reduces drastically by using these bounds, however the computational costs are enormous in the original version with the convex hull formulation (CH) and they are still substantial with the bundle approach (BD) from Gaar and Rendl (2019, 2020). Therefore, we now discuss the numerical results of the B&B algorithm using the new relaxations (VF) and (SH).

Looking at Table 6, the first thing we observe is that both (VF) and (SH) never lead to a MOSEK error, hence they are more robust than the other versions, presumably due to their smaller size of the SDPs to solve in the B&B nodes. For 9 instances both (VF) and (SH) and in 13 instances at least one of (VF) and (SH) is able to finish within the time limit for an instance where both (CH) and (BD) were not able to finish.

If we compare the number of B&B nodes in Table 6 for the finished instances, then we see that typically the number of B&B nodes for (VF) is smaller than those of (BD), which makes sense because in (BD) we only approximately solve (3) whereas in (VF) we solve a possibly very tight relaxation of (3) exactly. The number of B&B nodes needed by (SH) is typically a little bit larger than the one of (VF), which is in tune with the empirical finding that (5) gives better bounds than (8) in the small example considered in Sect. 2.5. In a nutshell, for finished runs typically (CH) and (VF) need roughly the same number of nodes, (SH) needs a little bit and (BD) needs many more nodes in the B&B tree.

As for the running times, in Fig. 1 we see that both (VF) and (SH) are faster than (BD) and considerably faster than (CH). (VF) is a little bit slower than (SH). For those instances that cannot be solved to optimality, the gap when the time limit is reached is roughly the same for (VF) and (SH), and it is considerably tighter than for (BD).

We have demonstrated that within a B&B algorithm both our relaxations (VF) and (SH) work better than already existing SDP based methods. In particular using (SH) allows to keep the majority of the strength of the upper bound (3) (i.e., keeping the number of vertices in the B&B tree low) by reducing the running time so that within the time limit almost 60% of the instances are solved, as compared to (CH) that only manages to solve a bit more than 20%.

## Conclusions

We introduced an algorithm for computing the stability number of a graph using semidefinite programming. While there exist several exact solution methods for finding the stability number, those based on semidefinite programming are rather rare.

We implemented a B&B algorithm using the SDP relaxations introduced in Gaar and Rendl (2019, 2020) together with heuristics from the literature. Moreover, we further relaxed the SDPs, getting more tractable SDPs still producing high-quality upper bounds. This is confirmed by the numerical experiments where we compare the number of nodes to be explored in the B&B tree as well as the CPU times.

While SDPs produce strong bounds, the computational expense for solving the SDPs is sometimes huge. In particular, there is potential for improvement of the running time for solving SDPs with many constraints. We use MOSEK as a solver, which uses the interior point method to solve an SDP. For large instances it would be beneficial to use a solver based on the boundary point method (Povh et al. 2006; Malick et al. 2009) or DADAL (De Santis et al. 2018). Moreover, the solver computing $\vartheta ^+$ as an upper bound (Cerulli et al. 2020) combined with the relaxations above, may push the performance of the B&B solver even further. Also, these other solvers are capable of doing warm starts, that can have big advantages within a B&B framework. Since all these implementations are available as MATLAB source code only, they need to be translated to C or  first. This will be part of our future work.

Another line of future research is working out different strategies for identifying violated subgraphs, that should also lead to a more efficient overall algorithm.

## Data Availability

All data comes from publicly available sources.

## Footnotes

1 Available at www-user.tu-chemnitz.de/~helmberg/rudy.tar.gz

## Code availability

The source code is available upon request from the authors.
